# Neonatal Marfan syndrome: a case report of a novel fibrillin 1 mutation, with genotype-phenotype correlation and brief review of the literature

**DOI:** 10.1186/s13052-024-01756-0

**Published:** 2024-09-18

**Authors:** Flaminia Pugnaloni, Domenico Umberto De Rose, Maria Cristina Digilio, Monia Magliozzi, Annabella Braguglia, Laura Valfrè, Alessandra Toscano, Andrea Dotta, Alessandra Di Pede

**Affiliations:** 1 Neonatal Intensive Care Unit, “Bambino Gesù” Children’s Hospital IRCCS, Rome, 00165 Italy; 2 Department of Pediatrics, Obstetrics and Gynecology, “Sapienza” University of Rome, Rome, 00161 Italy; 3 Medical Genetics Unit, “Bambino Gesù” Children’s Hospital IRCCS, Rome, 00165 Italy; 4 Translational Cytogenomics Research Unit, “Bambino Gesù” Children’s Hospital IRCCS, Rome, 00165 Italy; 5 Neonatal Sub-Intensive Care Unit and Follow-up, “Bambino Gesù” Children’s Hospital IRCCS, Rome, 00165 Italy; 6 Neonatal Surgery Unit, “Bambino Gesù” Children’s Hospital IRCCS, Rome, 00165 Italy; 7 Perinatal Cardiology Unit, “Bambino Gesù” Children’s Hospital IRCCS, Rome, 00165 Italy

**Keywords:** Neonatal Marfan syndrome, Cardiac failure, Phenotype-genotype correlation, Clinical genetics, Case report

## Abstract

**Background:**

Neonatal Marfan syndrome (nMFS) is a rare condition characterized by severe phenotype and poor prognosis. nMFS is caused by mutations in a specific region of the fibrillin 1 gene (FBN1). Prompt recognition of typical signs of neonatal presentation, such as characteristic facial anomalies with senile appearance, arthrogryposis, and campto-arachnodactyly, is fundamental for performing an early cardiological examination. This usually reveals rapidly progressive cardiovascular disease due to severe atrioventricular valve dysfunction.

**Case presentation:**

Herein, we report the case of an early-onset cardiac failure in a neonate with Marfan syndrome, with a brief review of the literature of cases with cardiac involvement in neonatal age. Clinical exome sequencing identified the novel heterozygous *de novo* missense variant c.3152T > G in FBN1 gene (NM_000138.4), causing the aminoacidic change p.Phe1051Cys. Phenotype-genotype correlation led to a multidisciplinary diagnostic and management workflow.

**Conclusion:**

The prompt recognition of a typical phenotype such as that of Marfan syndrome should lead to a detailed evaluation and close follow-up of cardiac morphology and function. Indeed, multi-disciplinary evaluation based on genotype-phenotype correlations of nMFS cases is essential to finding out the best medical and surgical approach, predicting the relevant impact on patient prognosis, and adequately counseling their families.

## Background

Neonatal Marfan syndrome (nMFS) is a rare condition characterized by severe phenotype and poor prognosis, caused by mutations in the specific “neonatal region” of the fibrillin 1 gene (FBN1) [1]. Prompt recognition of typical signs of neonatal presentation, such as characteristic facial anomalies with senile appearance, arthrogryposis, and campto-arachnodactyly, is fundamental for performing an early cardiological examination. This usually reveals rapidly progressive cardiovascular disease due to severe atrioventricular valve dysfunction.

Herein, we report the case of an early-onset cardiac failure in a neonate with Marfan syndrome, with a brief review of the literature of cases with cardiac involvement in neonatal age. Clinical exome sequencing revealed a *de novo* missense variant of the FBN1 gene. Phenotype-genotype correlation led to a multidisciplinary diagnostic and management workflow.

## Case presentation

A female neonate spontaneously conceived was born in a 2nd-level hospital at 39 weeks of gestational age (GA), to a 32-year-old primigravida through spontaneous delivery. Oligohydramnios and multiple complex choroid cysts were noticed in the last two weeks of pregnancy. No invasive prenatal testing was performed. Family history revealed a first-degree cousin (from the paternal side) affected by *de novo* Baraitser-Winter syndrome, and a paternal aunt whose pregnancy was interrupted because of a not-specified chromosomal disorder.

**Fig. 1:**
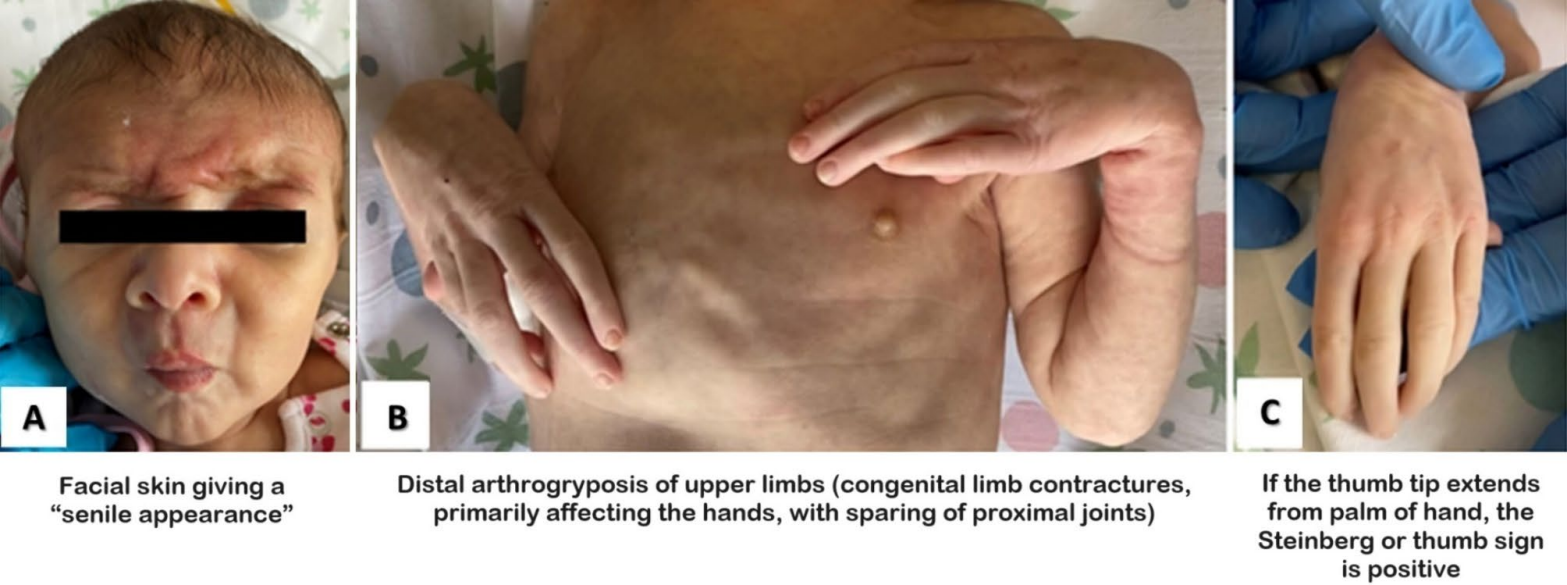
Dysmorphic features of our patient: **A**) senile facial appearance; **B**) distal arthrogryposis of upper limbs; **C**) severe arachnodactyly of hands and positive Steinberg sign (or thumb sign) of left hand

Apgar score was 8 and 9 at the 1st and 5th minutes, respectively. Birth weight was 2750 gr (16th centile, z-score: -1,01 SDS according to INeS charts [2]), length 49 cm (47th centile, z-score: -0.07 SDS), and head circumference 35 cm (86th centile, z-score: 1.06 SDS). At birth, several dysmorphic features were noticed, including brachycephaly, triangular and asymmetric face with a typical “senile” appearance (Fig. 1.A) and hypertelorism, down-slanted palpebral fissures, blepharophimosis, blue sclerae, anteverted nares, narrow mouth, micrognathia, and low-set ear. A distal arthrogryposis of the upper (Fig. 1.B) and lower limbs and severe arachnodactyly of hands (Fig. 1.C) and feet were evident. In particular, the Steinberg sign (well-known as the “thumb” sign) was positive in both hands (Fig. 1.C).

Cerebral ultrasound (CUS) performed within the first days of life confirmed multiple microcysts of the choroid plexus and increased echogenicity in the periventricular white matter. Brain magnetic resonance imaging (MRI) revealed a dysplastic appearance of cerebellar vermis and hemispheres, with a markedly thickened cerebellar cortex and loss of normal arborization of white matter. Hypoxic-ischemic lesions were noticed in the right frontal area. Chest computed tomography (CT), performed because of respiratory distress, showed a right posterolateral diaphragmatic relaxation with ipsilateral atelectasis of lung tissue. Skeletal X-ray showed no significant malformations. Echocardiography within the first 5 days of life yielded patent foramen ovale and patent ductus arteriosus (both with left-to-right shunt), mild tricuspid insufficiency, and moderate mitral insufficiency. A mild dilatation of the aortic bulb was also observed.

**Fig. 2:**
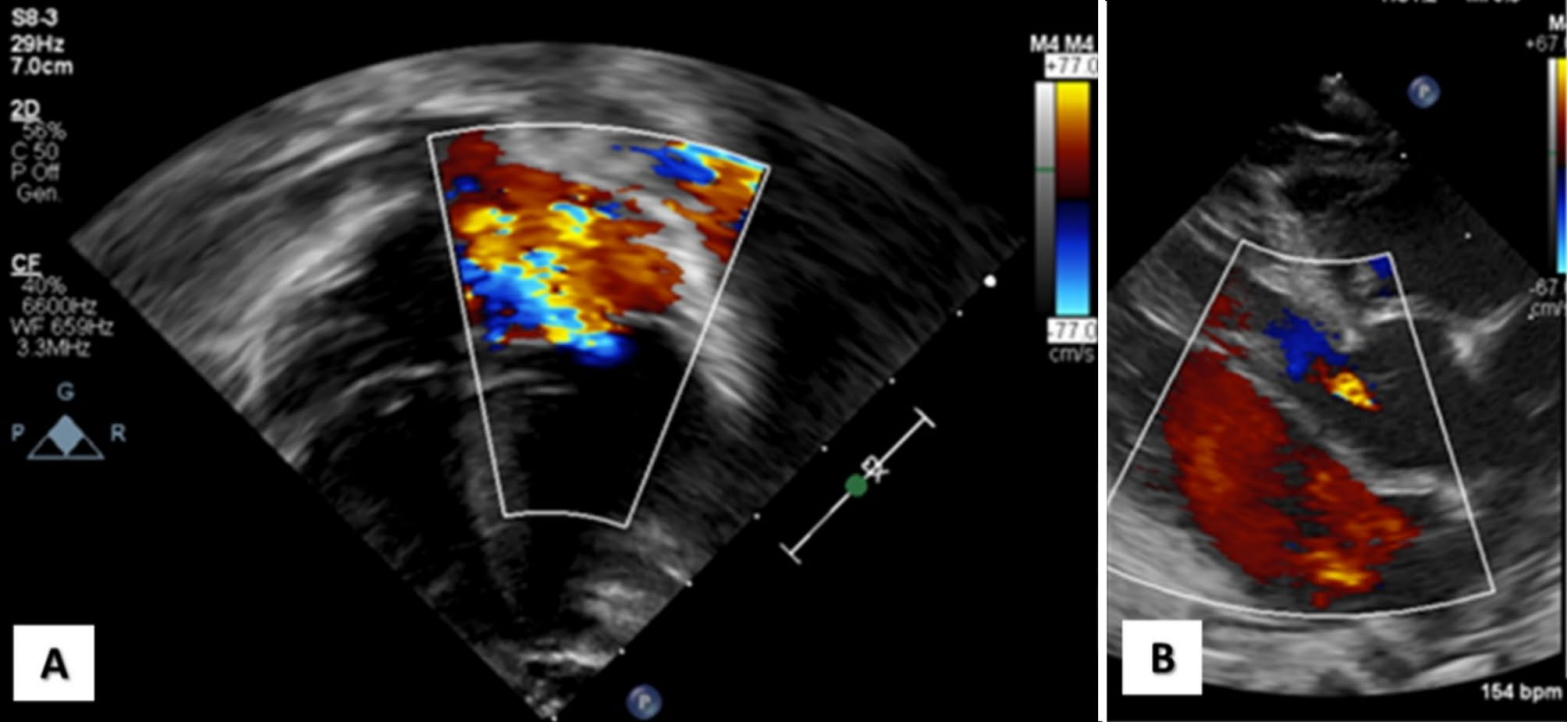
Cardiac involvement in our patient: **A**) severe mitral insufficiency from a 4-chamber view; **B**) aortic valve insufficiency from long axis view

The neonate was admitted on the 15th day of life to our 3rd-level children’s hospital to perform a specialistic evaluation. Physical examination revealed fair general conditions with polypnea and mild dyspnea; a 3/6 systolic murmur was audible. Cardiomegaly and dilation of the left ventricle were detected by echocardiography, with a mild-to-moderate biventricular dysfunction. A severely dysplastic mitral valve showed severe multi-jet insufficiency (Fig. 2.A), and aortic valve showed mild insufficiency (Fig. 2.B) and moderate dilation of Valsalva sinuses. The right ventricle was mildly dilated, with mild-to-moderate insufficiency of the tricuspid valve, and a dysplastic pulmonary valve with moderate insufficiency was also observed. The ophthalmological examination did not reveal pathological signs. Multivalvular involvement required initial conservative medical treatment using intravenous furosemide (up to 3 mg/kg) associated with oral spironolactone and captopril. Medical treatment led to a gradual decreasing trend of brain natriuretic peptide (BNP) and troponin values. The baby was discharged in her 5th month of life.

Blood karyotype and Chromosomal Microarray Analysis using platform Illumina^®^ CytoSNP 850k showed no pathogenic results. After these negative results, at 2 months of life, clinical exome analysis of trios was performed on DNA extracted from circulating leukocytes using kit Twist Custom Panel (Twist Bioscience) on the Illumina sequencing platform (NovaSeq6000, San Diego, CA). Next Generation Sequencing (NGS) analysis identified the novel heterozygous *de novo* missense variant c.3152T > G in FBN1 gene (NM_000138.4), causing the aminoacidic change p.Phe1051Cys. The variant was never described in the literature; it was not on the Genome Aggregation Database (gnomAD) and was classified as probably pathogenetic (class 4) according to guidelines of the American College of Medical Genetics and Genomics (ACMG) [3].

Subsequently, severe feeding intolerance and gastroesophageal reflux led to progressive malnutrition and a significant impact on children’s physical growth; at the 6th month of life, the infant was admitted again to our hospital and required nutrition support through the placement of a percutaneous endoscopic gastrostomy (PEG) tube. Despite maximal medical treatment and PEG placement, the infant progressively developed symptoms of congestive heart failure resulting from severe multivalvular insufficiency. At about 7 months of life, she underwent cardiosurgical intervention with mitral valvuloplasty and the use of annuloplasty, tricuspid, and pulmonary valvuloplasty.

Unfortunately, at 9 months and 19 days, the infant died of complications related to cardiac failure and severe malnutrition status (weight at death time 5070 gr, z-score: -3.04 according to WHO charts [4]).

## Methods

In order to review the literature about cardiac involvement in neonatal Marfan syndrome and compare other cases to ours, an extensive literature search in the MEDLINE database (via PubMed) has been performed up to December 31st, 2022. The following keywords, “neonatal,” “Marfan,” and “syndrome,” were searched as entree terms as well. All 239 retrieved articles of the last 20 years were screened, and then full texts of records deemed eligible for inclusion were assessed. References in the relevant papers were also reviewed. Papers written in languages other than English were excluded. Papers reporting a full description of the cases were included.

We systemically collected and summarized information on patients’ characteristics, cardiac involvement and procedures, and molecular findings.

## Results

A brief review of the literature on nMFS cases with cardiac involvement in the last 20 years is displayed in Table 1 [1, 5–28]. Beyond our case, we included other 27 cases, of whom three were born preterm. All 28 cases except one (reported by Postma et al.) had a birthweight greater than 2500 g. The mitral valve was involved in all cases, whereas the tricuspid valve was in 20/28 patients (71.4%). Aortic structures were involved in 23/28 cases (82.1%). Fifteen patients (53.6%) underwent cardiac procedures. FBN1 was the involved gene in all patients where a genetic diagnosis was available (21/28: 75%). Thirteen patients (46.4%) died at the time of writing.

**Table 1:** Brief review of nMFS cases in the last 20 years. NA: not available

Author, year	Sex	GA (weeks)	Delivery mode	Birthweight (centile) / Length (centile) / Head circumference (centile)	Mitral valve insufficiency / prolapse	Tricuspid valve insufficiency / prolapse	Aortic root dilation / Aortic valve regurgitation	Cardiac procedures	Molecular findings	Inheritation pattern	Age at last follow-up visit, outcome
Jacobs et al., 2002	F	At term	Vaginal delivery	2615 g (10th) / 52 cm (90th) / 32.5 cm (5th-10th)	Yes	Yes	Yes	Not performed	FBN1, T3276C, exon 24	NA	3.5 months, died
Shinawi et al., 2005	M	At term	Cesarean section	2880 g (25th) / 56 cm (> 95th) / NA	Yes	Yes	No	Balloon dilatation of pulmonary valve (4 days)	FBN1, IVS31-2 A > G, intron 31	NA	4.5 months, died
Ramaswamy et al., 2006	NA	At term	NA	3600 (75th) / 52 cm (75th)	Yes	Yes	Yes	Mitral valve repair (10 months)	NA	NA	15 months, alive
Sutherell et al., 2007	M	At term	NA	3300 (25th) / 51.5 cm (75th) / 34.5 cm (25th)	Yes	Yes	Yes	Medical treatment	FBN1, novel missense mutation, c.3256T > C, exon 26	De novo	4 days, died
Tekin et al., 2007	M	40	NA	3650 g (50th) / NA / NA	Yes	Yes	Yes	Medical treatment	NA	NA	3 months, died
Kochilas et al., 2008	F	37	Cesarean section	2900 g / NA / NA	Yes	Yes	Yes	Palliative care	FBN1, heterozygous 3256T > C, exon 26	De novo	69 days, died
Brito-Filho et al., 2013	M	At term	Cesarean section	3630 g / 51 cm / NA	Yes	Yes	Yes	Bentall-De Bono procedure (2 years), two mitral valve replacements (4 and 7 years)	NA	NA	7 years, alive
Šípek Jr. et al., 2014	F	40	Vaginal delivery	3670 (75th) / 53 cm (97th) / 34,5 (50th)	Yes	Yes	No	Medical treatment	FBN1, novel intronic c.4211-32_-13 del mutation, exon 35	Maternally inherited (mosaicism)	7 months, died
Elshershari & Harris, 2014	M	At term	NA	3200 g (25th) / 51 cm (50th) / NA	Yes	No	Yes	Medical treatment	FBN1, a heterozygous c.3959G. A, exon 31	Paternally inherited	3 months, alive
Amado et al., 2014	F	38	Cesarean section	3130 g (25th) / 46.5 cm (5-10th) / 35.5 cm (50-75th)	Yes	Yes	Yes	Mitral valvuloplasty (6 months)	FBN1, c.3458 G > A, exon 26	De novo	2 years and 10 months, alive
Ozyurt et al., 2015	F	39	Vaginal delivery	NA / NA / NA	Yes	Yes	Yes	Medical treatment	Not performed	NA	68 days, died
Bhutia et al., 2016	NA	At term	Vaginal delivery	NA / NA / NA	Yes	Yes	Yes	Medical treatment	Not performed	NA	3 months, died
Kitahara et al., 2016	M	38	Cesarean section	2295 g / 43.7 cm / NA	Yes	No	Yes	Mitral commissuroplasty (18 months), palliative procedure (3 years and 8 months)	NA	NA	11 years, alive
Le Gloan et al., 2016	F	39	Cesarean section	2950 g (-0.5 SD) / 50 cm (0 SD) / 33.5 cm (-1 SD)	Yes	Yes	Yes	Mitral and tricuspid valve annuloplasty (2 months)	FBN1, intronic mutation c.6163 + 3_6163 + 6del, exon 49	Maternally inherited	4 months, died
Maeda, 2016	M	40	NA	3768 g (90-97th) / 52.9 cm (> 97th) / NA	Yes	No	Yes	Medical treatment	FBN1, missense mutation (c. 3217 G > A), exon 26	NA	7 months, died
Maeda, 2016	F	37	Cesarean section	2850 g (25-50th) / NA / NA	Yes	Yes	No	Mitral and tricuspid valve annuloplasty (5 months); mitral valve replacement(12 months)	FBN1, exon-skipping mutation (c.IVS29 + 1G > A), exon 29	NA	22 months, died
Maeda, 2016	F	37	Cesarean section	2592 g (10-25th) / 50 cm (50-75th) / NA	Yes	Yes	No	Mitral and tricuspid valve annuloplasty (1 month)	FBN1, exon-skipping mutation (c.IVS30 + 1G > A), exon 30	NA	2 months, died
Peng et al., 2016	F	At term	Vaginal delivery	2600 g / NA / NA	Yes	Yes	Yes	NA	FBN1 missense mutation c.3331 T > C (p.Cys1111Arg), exon 26	De novo	8 months, alive
Heo et al., 2017	M	36	Vaginal delivery	3000 g (75-90th) / 54.5 cm (> 99th) / 35.5 cm (99th)	Yes	No	Yes	Surgical repair of rupture of mitral chordae (6 months)	FBN1, missense mutation (c. 3217 G > A), exon 26	NA	NA
Solé-Ribalta et al., 2019	NA	36	NA	NA / NA / NA	Yes	Yes	Yes	Palliative care	FBN1, heterozygous mutation (NM_000138.4): c.[3208 + 5G > A)	De novo	1 month, died
Tognato et al., 2019	M	At term	Cesarean section	3690 g (70th) / 56 cm (100th) / 36 cm (86th)	Yes	Yes	Yes	Mitral and tricuspidal valvuloplasty (11 months)	FBN1, heterozygous mutation c.3143 T > C (p.Ile1048Thr) w	NA	3 months, alive
Wojcik et al., 2019	NA	40	Vaginal delivery	NA	Yes	No	Yes	Aortic root replacement (3 months); mitral and tricuspid annuloplasty (12 months)	FBN1, heterozygous 385 bp deletion[GRCh38, exon 33	De novo	3 years, died
Veiga-Fernández et al., 2020	NA	37	Cesarean section	2530 g / NA / NA	Yes	No	No	Palliative care	NA	NA	3 months, died
Postma et al., 2021	F	32	Cesarean section	1710 g / NA / NA	Yes	Yes	Yes	Mitral annuloplasty and tricuspid commisuroplast (7 months)	FBN1, heterozygous splice site variant, IVS21 + 2T > C, exon15	Paternally inherited	14months, died
Yoon and Kong, 2021	M	40	Cesarean section	3560 g (75th) / 56.5 cm (90th) / 36 cm (90th)	Yes	No	No/Yes	Palliative care	FBN1, c.3964 + 1G > T, exon 32	NA	32 days, died
Motonaga et al., 2022	F	40	Vaginal delivery	3008 g / NA / NA	Yes	No	Yes	Mitral valve replacement (6 months)	FBN1,heterozygous missense variant (c.3379G > T), exon27	De novo	16 years, alive
Kawamura et al., 2022	M	38	Vaginal delivery	NA	Yes	Yes	No	Mitral and tricuspid valvuloplasty (6 months); mitral valve and tricuspid valve replacement (7 months)	FBN1, heterozygous missense variant of c.3706T > C (p. Cys1236Arg), exon 29	NA	13 months, alive

## Discussion and conclusion

We compared the severe cardiac involvement of a neonate with Marfan syndrome to the cases available in the literature. Cardiac involvement is the main determinant in the prognosis of neonates with Marfan syndrome, and it is usually life-threatening. Severe valvular disease affecting mostly mitral and tricuspid valves quickly progresses to congestive heart failure and premature death within the first 2 years of life [1, 21, 22, 29, 30]. Indeed, cardiac involvement in neonates is usually characterized by severe multivalvular insufficiency instead of aortic structures involvement, which is a typical feature in adults and older children [31, 32].

Congenital pulmonary emphysema is also often detected in nMFS [33]. Rarely, patients with nMFS may develop progressive but not fatal heart failure; some young adults have been described [34].

Molecular analysis shows that most nMFS mutations are sporadic and occur in the so-called “neonatal region” of *FBN1* gene mapping between exons 24 and 32 [35, 36].

In our case, we identified a novel missense variant, p.Phe1051Cys, located in exon 26 of the FBN1 gene inside the “neonatal critical region” that was not previously reported in the literature.

Interesting genotype-phenotype associations for both cardiovascular and extra-cardiovascular manifestations were identified in the pediatric population [37]. Previous studies and case reports demonstrated that mutations located in FBN1 “neonatal region” are usually associated with a rapidly worsening cardiac disease, poor response to medications [36], and severe congestive heart failure, which represent the main cause of early death. In particular, patients carrying variants in a specific region (amino acids 1028–1088, corresponding to exon 25 and a few residues from exon 26) show a worse prognosis with heart failure-related death within the first year of life, irrespective of gender [38].

Early genotype analysis and prompt phenotype recognition can potentially drive accurate genetic counseling. Indeed, the prompt recognition of a typical phenotype such as that of Marfan syndrome should lead to a detailed evaluation and close follow-up of cardiac morphology and function. Timely diagnosis is increasingly important in looking for genotype-phenotype characterization and improving early therapeutic strategies.

Despite their low quantity in skeletal matrices, MFS causes severe skeletal defects, highlighting the importance of fibrillin-1 and microfibrils in bone formation and function [39]. The extent of musculoskeletal disease is quite significant in older patients with Marfan syndrome: scoliosis, pectus deformity, and deformity of the foot. Many will need a specific follow-up, requiring corrective surgery during their life span [40].

Similarly, fibrillin-1-containing microfibrils are ubiquitous in the normal eye. Ectopia lentis in MFS patients is likely caused by an FBN1 mutation that prevents fibrillin-1 production. If they survive, MFS patients experience different ocular features depending on the mutation and severity of the illness. Patients with MFS typically acquire lenticular and/or axial myopia before the age of ten and should see an ophthalmologist to examine their near-sightedness [39].

Multi-disciplinary evaluation based on genotype-phenotype correlations of nMFS cases is essential to determine the best medical and surgical approach, predict the relevant impact on patient prognosis, and adequately counsel their families. MFS is an example of a syndrome where an early personalized approach to address a dynamic, genetically determined condition can make a difference in outcome [41].

In light of this, a careful evaluation of all clinical signs by neonatologists is mandatory: in particular, the Steinberg sign (also known as the thumb sign) presence should be considered a potential handle sign for diagnostic suspicion, and every neonatologist should rule out Marfan syndrome in cases like this. This can be useful to perform a correct differential diagnosis, like, for example, with distal arthrogryposis syndromes or other congenital defects with cardiac involvement, with the aim of timely reaching the correct diagnosis [42, 43].

The current databases should be updated with the genomic and phenotypic findings of the present patient in order to provide a better characterization of such a rare disease. Additional patients and the identification of new mutations will increase the knowledge of the molecular bases and the pathogenic mechanisms underlying Marfan syndrome with neonatal onset.

Finally, clinicians must be aware of the possibility that neonates may have a severely poor outcome, even in the absence of symptoms in the first weeks of life.

## Data Availability

All data generated or analysed during this study are included in this published article.

## Abbreviations

ACMG: American College of Medical Genetics
CT: Computed tomography
CUS: Cerebral ultrasound
FBN1: Fibrillin 1
GA: Gestational age
gnomAD: Genome Aggregation Database
MRI: Magnetic resonance imaging
NGS: Next Generation Sequencing
nMFS: Neonatal Marfan syndrome
PEG: Percutaneous endoscopic gastrostomy
SDS: Standard deviation
WHO: World Health Organization

## References

[1] Amado M, Calado MA, Ferreira R, Lourenço T. Neonatal Marfan syndrome: a successful early multidisciplinary approach. Case Rep. 2014;bcr2013202438. 10.1136/bcr-2013-202438 10.1136/bcr-2013-202438 PMC4069814 24928929

[2] Bertino E, Spada E, Occhi L, Coscia A, Giuliani F, Gagliardi L, Gilli G, Bona G, Fabris C, De Curtis M, Milani S. Neonatal anthropometric charts: the Italian neonatal study compared with other European studies. J Pediatr Gastroenterol Nutr. 2010;51:353–61. 10.1097/MPG.0b013e3181da213e 20601901 10.1097/MPG.0b013e3181da213e

[3] Richards S, Aziz N, Bale S, Bick D, Das S, Gastier-Foster J, Grody WW, Hegde M, Lyon E, Spector E, Voelkerding K, Rehm HL, ACMG Laboratory Quality Assurance Committee. Standards and guidelines for the interpretation of sequence variants: a joint consensus recommendation of the American College of Medical Genetics and Genomics and the Association for Molecular Pathology. Genet Med. 2015;17(5):405–24. 10.1038/gim.2015.30 25741868 10.1038/gim.2015.30 PMC4544753

[4] The WHO Child Growth Standards. https://www.who.int/tools/child-growth-standards/standards. (Accessed 11 Jan 2023).

[5] Jacobs AM, Toudjarska I, Racine A, Tsipouras P, Kilpatrick MW, Shanske A. A recurring FBN1 gene mutation in neonatal Marfan syndrome. Arch Pediatr Adolesc Med. 2002;156(11):1081–5. 10.1001/archpedi.156.11.1081 12413333 10.1001/archpedi.156.11.1081

[6] Shinawi M, Boileau C, Brik R, Mandel H, Bentur L. Splicing mutation in the fibrillin-1 gene associated with neonatal Marfan syndrome and severe pulmonary emphysema with tracheobronchomalacia. Pediatr Pulmonol. 2005;39(4):374–8. 10.1002/ppul.20174 15666366 10.1002/ppul.20174

[7] Ramaswamy P, Lytrivi ID, Nguyen K, Gelb BD. Neonatal Marfan syndrome: in utero presentation with aortic and pulmonary artery dilatation and successful repair of an acute flail mitral valve leaflet in infancy. Pediatr Cardiol. 2006;27(6):763–5. 10.1007/s00246-006-1378-0 10.1007/s00246-006-1378-0 17091324

[8] Sutherell J, Zarate Y, Tinkle BT, Markham LW, Cripe LH, Hyland JC, Witte D, Hopkin RJ, Hinton RB. Novel fibrillin 1 mutation in a case of neonatal Marfan syndrome: the increasing importance of early recognition. Congenit Heart Dis. 2007;2(5):342–6. 10.1111/j.1747-0803.2007.00123.x 10.1111/j.1747-0803.2007.00123.x 18377451

[9] Tekin M, Cengiz FB, Ayberkin E, Kendirli T, Fitoz S, Tutar E, Ciftçi E, Conba A. Familial neonatal Marfan syndrome due to parental mosaicism of a missense mutation in the FBN1 gene. Am J Med Genet A. 2007;143A(8):875–80. 10.1002/ajmg.a.31660 17366579 10.1002/ajmg.a.31660

[10] Kochilas L, Gundogan F, Atalay M, Bliss JM, Vatta M, Pena LS, Abuelo D. A novel mutation of the fibrillin-1 gene in a newborn with severe Marfan syndrome. J Perinatol. 2008;28(4):303–5. 10.1038/sj.jp.7211915 18379569 10.1038/sj.jp.7211915

[11] Brito-Filho SL, Oporto V, Campos O, Alvares AB, Carvalho AC. A case of neonatal Marfan syndrome with good late follow-up: is it possible to avoid an early unfavourable outcome? Cardiol Young. 2013;23(2):301–3. 10.1017/S104795111200090X 22813538 10.1017/S104795111200090X

[12] Sípek A Jr, Grodecká L, Baxová A, Cibulková P, Dvořáková M, Mazurová S, Magner M, Zeman J, Honzík T, Freiberger T. Novel FBN1 gene mutation and maternal germinal mosaicism as the cause of neonatal form of Marfan syndrome. Am J Med Genet A. 2014;164A(6):1559–64. 10.1002/ajmg.a.36480 24668922 10.1002/ajmg.a.36480

[13] Elshershari H, Harris C. Paternal fibrillin-1 mutation transmitted to an affected son with neonatal marfan syndrome: the importance of early recognition. Cardiol Young. 2014;24(4):735–8. 10.1017/S1047951113001029 23930893 10.1017/S1047951113001029

[14] Ozyurt A, Baykan A, Argun M, Pamukcu O, Halis H, Korkut S, Yuksel Z, Gunes T, Narin N. Early onset marfan syndrome: atypical clinical presentation of two cases. Balkan J Med Genet. 2015;18(1):71–6. 10.1515/bjmg-2015-0008 26929908 10.1515/bjmg-2015-0008 PMC4768828

[15] Buthia E, Kumar P, Kishore S, Yadav DK. Unremitting congestive heart failure: neonatal Marfan syndrome. J Clin Neonatol. 2016;5(2):128–30.

[16] Kitahara H, Aeba R, Takaki H, Shimizu H. Palliative mitral valve repair during infancy for neonatal Marfan syndrome. Ann Thorac Surg. 2016;101(5):1987–8. 10.1016/j.athoracsur.2015.06.115 27106438 10.1016/j.athoracsur.2015.06.115

[17] Le Gloan L, Hauet Q, David A, Hanna N, Arfeuille C, Arnaud P, Boileau C, Romefort B, Benbrik N, Gournay V, Joram N, Baron O, Isidor B. Neonatal Marfan Syndrome: report of a case with an inherited splicing mutation outside the neonatal domain. Mol Syndromol. 2016;6(6):281–6. 10.1159/000443867 27022329 10.1159/000443867 PMC4802997

[18] Maeda J, Kosaki K, Shiono J, Kouno K, Aeba R, Yamagishi H. Variable severity of cardiovascular phenotypes in patients with an early-onset form of Marfan syndrome harboring FBN1 mutations in exons 24–32. Heart Vessels. 2016;31(10):1717–23. 10.1007/s00380-016-0793-2 26796135 10.1007/s00380-016-0793-2

[19] Peng Q, Deng Y, Yang Y, Liu H. A novel fibrillin-1 gene missense mutation associated with neonatal Marfan syndrome: a case report and review of the mutation spectrum. BMC Pediatr. 2016;16:60. 10.1186/s12887-016-0598-6 27138491 10.1186/s12887-016-0598-6 PMC4852411

[20] Heo JS, Song JY, Choi EY, Kim EH, Kim JH, Park SE, Jeon JH. Atypical neonatal Marfan syndrome with p.Glu1073Lys mutation of FBN1: the first case in Korea. J Korean Med Sci. 2017;32(1):1–3. 10.3346/jkms.2017.32.1.1 27914124 10.3346/jkms.2017.32.1.1 PMC5143279

[21] Solé-Ribalta A, Rodríguez-Fanjul X, Carretero-Bellon JM, Pascual-Sala C, Martorell-Sampol L, Bobillo-Pérez S, Morillo-Palomo AM. Neonatal Marfan syndrome: a rare, severe, and life-threatening genetic disease. J Pediatr. 2019;211:221–e2212. 10.1016/j.jpeds.2019.03.033 31053350 10.1016/j.jpeds.2019.03.033

[22] Tognato E, Perona A, Aronica A, Bertola A, Cimminelli L, De Vecchi S, Eshraghy MR, Loperfido B, Vivenza C, Manzoni P. Neonatal Marfan syndrome. Am J Perinatol. 2019;36(02):S74–6. 10.1055/s-0039-1691770 31238364 10.1055/s-0039-1691770

[23] Wojcik MH, Thiele K, Grant CF, Chao K, Goodrich J, O’Donnell-Luria A, Lacro RV, Tan WH, Agrawal PB. Genome sequencing identifies the pathogenic variant missed by prior testing in an infant with Marfan Syndrome. J Pediatr. 2019;213:235–40. 10.1016/j.jpeds.2019.05.029 31235381 10.1016/j.jpeds.2019.05.029 PMC6765408

[24] Veiga-Fernández A, Joigneau Prieto L, Álvarez T, Ruiz Y, Pérez R, Gámez F, Ortega Abad V, Yllana F, De León-Luis J. Perinatal diagnosis and management of early-onset Marfan syndrome: case report and systematic review. J Matern Fetal Neonatal Med. 2020;33(14):2493–504. 10.1080/14767058.2018.1552935 30652519 10.1080/14767058.2018.1552935

[25] Postma JK, Altamirano-Diaz L, Rupar CA, Siu VM. Symptomatic mosaicism for a novel FBN1 splice site variant in a parent causing inherited neonatal Marfan syndrome. Am J Med Genet A. 2021;185(8):2507–13. 10.1002/ajmg.a.62339 33988295 10.1002/ajmg.a.62339

[26] Yoon SH, Kong Y. Severe neonatal Marfan syndrome with a novel mutation in the intron of the FBN1 gene: a case report. Med (Baltim). 2021;100(6):e24301. 10.1097/MD.0000000000024301 10.1097/MD.0000000000024301 PMC10545169 33578525

[27] Motonaga T, Ohnishi Y, Okada S, Suzuki Y, Furuta T, Kawamura M, Okayama N, Suehiro Y, Hasegawa S. Successful mitral valve replacement in an infant with neonatal Marfan syndrome due to a novel missense mutation of the FBN1 gene. Int Heart J. 2022;63(4):777–81. 10.1536/ihj.21-821 35831148 10.1536/ihj.21-821

[28] Kawamura J, Ueno K, Kawano Y. Neonatal Marfan syndrome with missense variant of c.3706T > C undergoing bilateral atrioventricular valve replacement. Cardiol Young. 2022;32(5):833–6. 10.1017/S1047951121003905 34526162 10.1017/S1047951121003905

[29] Heide Hter, Schrander- Stumpel CTRM, Pals G, Delhaas T. Neonatal Marfan syndrome: clinical report and review of the literature. Clin Dysmorphol. 2005;14:81–4. 15770129

[30] Hennekam RCM. Severe infantile Marfan syndrome versus neonatal Marfan syndrome. Am J Med Genet A. 2005;139A:1–1. 10.1002/ajmg.a.30979 10.1002/ajmg.a.30979 16222685

[31] Ekhomu O, Naheed ZJ. Aortic involvement in pediatric Marfan syndrome: a review. Pediatr Cardiol. 2015;36(5):887–95. 10.1007/s00246-015-1101-0 25669767 10.1007/s00246-015-1101-0

[32] Nucera M, Heinisch PP, Langhammer B, Jungi S, Mihalj M, Schober P, Luedi MM, Yildiz M, Schoenhoff FS. The impact of sex and gender on aortic events in patients with Marfan syndrome. Eur J Cardiothorac Surg. 2022;62(5):ezac305. 10.1093/ejcts/ezac305 35543473 10.1093/ejcts/ezac305

[33] Stheneur C, Faivre L, Collod-Béroud G, Gautier E, Binquet C, Bonithon-Kopp C, Claustres M, Child AH, Arbustini E, Adès LC, Francke U, Mayer K, Arslan-Kirchner M, De Paepe A, Chevallier B, Bonnet D, Jondeau G, Boileau C. Prognosis factors in probands with an FBN1 mutation diagnosed before the age of 1 year. Pediatr Res. 2011;69:265–70. 10.1203/PDR.0b013e3182097219 21135753 10.1203/PDR.0b013e3182097219

[34] Hussain S, Geddes G, Darragh R, Parent JJ. Successful heart transplantation in a patient with neonatal Marfan syndrome. J Heart Lung Transpl. 2022;41:S515. 10.1016/j.healun.2022.01.1306

[35] Booms P, Cisler J, Mathews KR, Godfrey M, Tiecke F, Kaufmann UC, Vetter U, Hagemeier C, Robinson PN. Novel exon skipping mutation in the fibrillin-1 gene: two ‘hot spots’ for the neonatal Marfan syndrome. Clin Genet. 1999;55:110–7. 10.1034/j.1399-0004.1999.550207.x 10189088 10.1034/j.1399-0004.1999.550207.x

[36] Tiecke F, Katzke S, Booms P, Robinson PN, Neumann L, Godfrey M, Mathews KR, Scheuner M, Hinkel GK, Brenner RE, Hövels-Gürich HH, Hagemeier C, Fuchs J, Skovby F, Rosenberg T. Classic, atypically severe and neonatal Marfan syndrome: twelve mutations and genotype-phenotype correlations in FBN1 exons 24–40. Eur J Hum Genet. 2001;9:13–21. 10.1038/sj.ejhg.5200582 11175294 10.1038/sj.ejhg.5200582

[37] Meester JAN, Peeters S, Van Den Heuvel L, Vandeweyer G, Fransen E, Cappella E, Dietz HC, Forbus G, Gelb BD, Goldmuntz E, Hoskoppal A, Landstrom AP, Lee T, Mital S, Morris S, Olson AK, Renard M, Roden DM, Singh MN, Selamet Tierney ES, Tretter JT, Van Driest SL, Willing M, Verstraeten A, Van Laer L, Lacro RV, Loeys BL. (2022) Molecular characterization and investigation of the role of genetic variation in phenotypic variability and response to treatment in a large pediatric Marfan syndrome cohort. Genet Med. 2022;24:1045–1053. 10.1016/j.gim.2021.12.015 10.1016/j.gim.2021.12.015 PMC9680912 35058154

[38] Brogger MN, Fernandez Ferro G, Cardenas Reyes I, Ochoa JP, Garcia Hernandez S, Valverde M, Fernandez X, Garcia Giustiniani D, Lamounier A, De La Higuera Romero L, Ortiz Genga M, Monserrat L, McKenna WJ. Narrowing of the neonatal region in the FBN1 gene. Eur Heart J. 2021;42:ehab7241988. 10.1093/eurheartj/ehab724.1988

[39] Milewicz DM, Braverman AC, De Backer J, Morris SA, Boileau C, Maumenee IH, Jondeau G, Evangelista A, Pyeritz RE. Marfan syndrome. Nat Rev Dis Primers. 2022;8(1):3. 10.1038/s41572-022-00338-w 35039531 10.1038/s41572-022-00338-w

[40] Andersen NH, Hauge EM, Baad-Hansen T, Groth KA, Berglund A, Gravholt CH, Stochholm K. Musculoskeletal diseases in Marfan syndrome: a nationwide registry study. Orphanet J Rare Dis. 2022;17(1):118. 10.1186/s13023-022-02272-2 35248143 10.1186/s13023-022-02272-2 PMC8898450

[41] Baban A, Parlapiano G, Cicenia M, Armando M, Franceschini A, Pacifico C, Panfili A, Zinzanella G, Romanzo A, Fusco A, Caiazza M, Perri G, Galletti L, Digilio MC, Buonuomo PS, Bartuli A, Novelli A, Raponi M, Limongelli G. Unique features of cardiovascular involvement and progression in children with Marfan syndrome justify dedicated multidisciplinary care. J Cardiovasc Dev Dis. 2024;11(4):114. 10.3390/jcdd11040114 38667733 10.3390/jcdd11040114 PMC11050181

[42] Serra G, Antona V, Cannata C, Giuffrè M, Piro E, Schierz IAM, Corsello G. Distal arthrogryposis type 5 in an Italian family due to an autosomal dominant gain-of-function mutation of the PIEZO2 gene. Ital J Pediatr. 2022;48(1):133. 10.1186/s13052-022-01329-z 35906671 10.1186/s13052-022-01329-z PMC9336156

[43] Serra G, Felice S, Antona V, Di Pace MR, Giuffrè M, Piro E, Corsello G. Cardio-facio-cutaneous syndrome and gastrointestinal defects: report on a newborn with 19p13.3 deletion including the MAP 2 K2 gene. Ital J Pediatr. 2022;48(1):65. 10.1186/s13052-022-01241-6 35509048 10.1186/s13052-022-01241-6 PMC9069788

